# PReS-FINAL-2113: The association of the NFKB1 gene polymorphism with systemic onset juvenile idiopathic arthritis in Russia

**DOI:** 10.1186/1546-0096-11-S2-P125

**Published:** 2013-12-05

**Authors:** VA Malievsky, TV Viktorova, KV Danilko, LS Nazarova

**Affiliations:** 1 Department of Hospital Pediatrics, Bashkir State Medical University, Ufa, Russian Federation; 2 Biology Department, Laboratory of Human Physiological Genetics, Bashkir State Medical University, Institute of Biochemistry and Genetics, Ufa, Russian Federation; 3 Biology Department, Central Scientific Research Laboratory, Ufa, Russian Federation; 4 Biology Department, Bashkir State Medical University, Ufa, Russian Federation

## Introduction

Juvenile idiopathic arthritis (JIA) is a childhood onset autoimmune disorder characterized by inflammation of joints and other tissues. The basis for susceptibility to common autoimmune and inflammatory diseases, including JIA, is a complex interplay between multiple genetic and environmental risk factors. NF-κb appears to be a central player in several autoimmune diseases, according to recent studies of genetic defects in autoreactive lymphoid cells in both murine models of autoimmunity and humans with diverse forms of autoimmunity. Meta-analysis suggested a possible association between NFKB1 -94 ins/delattg promoter polymorphism and certain autoimmune and inflammatory diseases in the Asian population, but not in the Caucasian population.

## Objectives

The aim of the study was to investigate the NFKB1 -94 ins/del ATTG polymorphism (rs28362491) in relation to risk of different forms of JIA in patients from Russia.

## Methods

The NFKB1-94 ins/del ATTG genotypes were evaluated by using PCR method. A total of 155 JIA patients and 145 healthy controls from Bashkortostan, Russia were successfully investigated. Patients were diagnosed according to the ILAR criteria. There were 63 persistent oligoarthritis (PO), 48 rheumatoid factor-negative polyarthritis (RFNP), 18 systemic onset (SO) JIA and 26 other subtypes patients. Chi-square (χ^2^) test and odds ratio (OR) were estimated to evaluate the association.

## Results

Allele and genotype occurrence were in Hardy Weinberg Equilibrium in both groups. No differences were observed in allele and genotype frequencies of the NFKB1 -94 ins/del ATTG polymorphism between total JIA patients and controls. Stratification analysis across all ILAR subgroups revealed only weak association of the ins*ins* genotype with SO in comparison to all others JIA types (χ2 = 4,014, p = 0,045, OR = 3,063, 95% CI 1,127-8,324). In contrast frequency of the ins*del* genotype was significantly lower in SO groups than both in controls and all others JIA patients (χ2 = 6,824, p = 0,010, OR = 0,182, 95% CI 0,051-0,656; χ2 = 6,682, p = 0,011, OR = 0,184, 95% CI 0,051-0,664).

## Conclusion

These findings demonstrate low risk of the systemic onset JIA in ins*del* genotype of the NFKB1 -94 ins/del polymorphism carriers from Bashkortostan, Russia, and high risk of the systemic onset JIA patients with ins*ins* genotype.

## Disclosure of interest

None declared.

